# Synergistic effect of cement mortar types and encapsulated healing agents on crack healing in sustained notched concrete beams subjected to flexural loads

**DOI:** 10.1038/s41598-025-25912-6

**Published:** 2025-11-18

**Authors:** Rasha A. El-Sadany, Hossam El-Din M. Sallam, Mohamed A. R. Elmahdy

**Affiliations:** 1 Radiation Engineering Department, National Center for Radiation Research and Technology, Egyptian Atomic Energy Authority, Cairo, Egypt; 2 Materials Engineering Department, Faculty of Engineering, Zagazig University, Zagazig, 44519 Egypt; 3 Civil Engineering Department, Misr Higher Institute of Eng.&Tech, Mansoura, Egypt

**Keywords:** Sustainability, Self-healing concrete, Crack healing efficiency, Encapsulation technology, Expansive minerals, Microstructure characterization, Engineering, Materials science

## Abstract

Reinforced concrete structures have a critical durability challenge due to crack formation, facilitating moisture penetration, steel corrosion, and progressive structural deterioration. To address these challenges, this work systematically investigates the synergistic effects of pozzolanic materials in cement mortar and encapsulated healing agents on the autonomous repair capability of sustainable concrete notched beams under flexural loading. In this work, an experimental program consisting of three types of cement mortar with dimensions of 50 × 50 × 220 mm, it was performed using advanced material systems involving pozzolanic additives (silica fume (SF), marble powder (MP) and an encapsulated system incorporating expansive minerals (MgO, CaO, and bentonite) in macro-capsules with a thickness of 0.45 mm, a length of 50 mm, and inner diameters of 6.15 mm and 11.4 mm for the inner and outer capsules, within optimized mortar matrices. Healing efficiency was evaluated via crack area efficiency and load recovery coefficient measurements. Also, advanced microstructure characterization techniques (SEM–EDS, XRD, FT-IR) provided multiscale analysis of healing products and matrix-capsule interfaces to assess healing efficiency. The results showed that SF had superior pozzolanic and healing performance, achieving 87.5% crack sealing and 20% load recovery through increased pozzolanic activity and CSH production. Furthermore, microstructural analyses (SEM/XRD/FT-IR) validated its activity in matrix densification, CH reduction, and interfacial refinement. However, MP mostly acts as an inert filler, revealing the vital performance of SF for self-healing mortar systems. These findings contribute to developing autonomous self-healing technology, providing concrete with an extended service life for sustainable construction applications.

## Introduction

Concrete is the most widely used construction material globally, lauded for its strength, versatility, and affordability. However, its long-term performance is hampered by inherent weaknesses, particularly its susceptibility to cracking. Cracks, even microscopic ones, allow aggressive agents like water, chlorides, and carbon dioxide to ingress, leading to internal degradation and corrosion of steel reinforcement. This deterioration necessitates costly repairs, reduces structural integrity, and ultimately shortens the lifespan of concrete structures^1–4^. Traditional repair methods are often disruptive, time-consuming, and expensive. Moreover, they usually fail to address the underlying cause of the problem, leading to recurring repairs and an unsustainable cycle of degradation. This necessitates a paradigm shift in concrete technology, where materials can autonomously heal damage and maintain their functionality over time^5–7^

Self-healing concrete (SHC) emerges as a revolutionary solution to these challenges. By incorporating various healing mechanisms, SHC can automatically repair cracks and mitigate the detrimental effects of deterioration^8,9^. This enhances the durability and lifespan of structures and reduces maintenance costs and environmental impact. In light of growing infrastructure demands and sustainability concerns, the development and implementation of SHC technologies are crucial to ensuring our built environment’s long-term performance and resilience^10–13^. SHC is a cementitious material that can autonomously repair damage or cracks through intrinsic or extrinsic mechanisms, extending its service life and enhancing its durability^14,15^. SHC is a concrete that can automatically repair cracks and defects through built-in healing mechanisms, including but not limited to microcapsules, vascular networks, and biomineralization^16,17^.

Also, SHC provides a solution to address durability issues by autonomously repairing cracks and damage. Autogenous or autogenic healing is a notable type of self-healing in concrete, occurring through the internal reaction of unhydrated cement particles with water, producing additional calcium silicate hydrate (C–S–H) and calcium hydroxide to fill microcracks. This process is intrinsic to the concrete matrix and operates without external intervention, allowing for the closure of small cracks over time^18,19^. Autonomic healing is another prominent type wherein healing agents, such as encapsulated bacteria or polymers, are embedded within the concrete. When cracks form, these capsules rupture, releasing the healing agents, which react with environmental conditions to mend the damaged areas. This method provides a more active and targeted approach to healing, addressing a wider range of crack sizes and extending the service life of concrete structures^20,21^. Autogenous or autogenic healing methods in concrete represent an innovative avenue for autonomously repairing cracks and improving the durability of structures. One prevalent autogenic healing method involves using expansive agents, such as expansive types of cement or expansive minerals, which react with moisture and unhydrated cement particles to generate additional hydration products, filling cracks and enhancing the material’s mechanical properties. This process occurs naturally within the concrete matrix, contributing to crack closure and promoting the overall sustainability of concrete structures^22,23^.

Another approach to autogenic healing involves using supplementary cementitious materials (SCMs) like fly ash or slag. These SCMs react with calcium hydroxide and water to produce additional C–S–H gel, filling cracks and improving the material’s overall performance. Autogenic healing through SCMs not only addresses crack-related issues but also enhances the strength and durability of concrete, showcasing a versatile and sustainable strategy for long-lasting infrastructure^24–26^. In SHC, the distinctions between capsules, macrocaps, and microcaps are crucial for tailoring effective healing strategies to the specific needs and scale of concrete structures. Capsules, typically small containers embedded in concrete, house healing agents, such as bacteria or polymers, which are released upon rupture when cracks develop, initiating the repair process^27–29^. Macro-capsules, larger counterparts to capsules, are designed to hold a greater volume of healing agents, enhancing the potential for extensive crack healing, especially in larger fractures, and contributing to the overall durability of the concrete structure^30,31^.

On the other hand, microcaps are even smaller and focus on targeted healing in microcracks, allowing for precise placement within the concrete matrix. Their diminutive size is advantageous in addressing finer cracks that may escape detection in the early stages of formation. Understanding the differences in these capsule types is crucial for tailoring self-healing strategies to the specific needs and scale of concrete structures^27,32,33^. Macro capsules in SHC are essential in augmenting the healing process within larger fractures. These larger containers are designed to encapsulate a substantial volume of healing agents, such as bacteria or polymers, allowing for a more extensive coverage of cracks. The mechanism involves the rupture of macro capsules when cracks develop in the concrete matrix, releasing the healing agents into the damaged areas. Once released, these agents react with environmental conditions to form new mineral deposits or polymer structures, effectively closing the larger fractures and restoring the structural integrity of the concrete. Using macro capsules provides a robust solution for addressing significant cracks and contributes significantly to concrete structures’ overall durability and longevit^34–36^. Materials employed in capsules for SHC are meticulously chosen to align with the requirements of the concrete matrix and the desired healing mechanisms. Among the commonly utilized materials are polymers, such as polyurethane and epoxy, renowned for their versatility and ability to encapsulate healing agents effectively^37,38^. The continuous evolution of SHC technology has prompted the exploration of innovative materials. Bio-based polymers derived from renewable sources have emerged as environmentally friendly alternatives for capsule design, contributing to sustainable construction practices^39^. Furthermore, integrating smart materials, such as pH-responsive polymers or shape-memory polymers, introduces a level of sophistication in capsule functionality. These materials enable the controlled and targeted release of healing agents in response to specific environmental or structural conditions, enhancing the adaptability and efficiency of self-healing concrete systems^39,40^.

Materials incorporated into capsules for self-healing concrete, such as magnesium oxide (MgO), calcium oxide (CaO), and bentonite, have been extensively researched for their diverse properties and significant contributions to enhancing the durability of concrete structures. MgO, known for its expansive nature, is commonly integrated into capsules to facilitate crack sealing within the concrete matrix^41,42^. The expansive behavior of MgO actively participates in the autogenous healing process, contributing to the overall resilience of concrete structures and extending their service life^43,44^. Calcium oxide (CaO) plays a crucial role in self-healing concrete when utilized in capsules, particularly in carbonation. Upon reacting with carbon dioxide (CO2), CaO transforms into calcium carbonate (CaCO3), a mineral that precipitates and effectively fills cracks within the concrete structure. This carbonation-induced healing mechanism addresses crack-related issues and enhances the overall durability of concrete structures, contributing to their prolonged service life^44,45^. Bentonite, a natural clay mineral with exceptional swelling properties, has been found to have applications in capsule materials for self-healing concrete. When exposed to water, bentonite undergoes swelling, forming a gel-like substance that adeptly seals cracks and hinders the ingress of harmful substances into the concrete matrix. Incorporating bentonite into capsules establishes a protective barrier within cracks, actively supporting the self-healing process of concrete structures and improving their overall performance^46,47^.

This research addresses a critical challenge in concrete durability by developing an innovative self-healing system that combines pozzolanic materials (silica fume, marble powder) and encapsulated healing agents (MgO/CaO/bentonite). Also, the study provides a sustainable solution for enhanced crack sealing and load recovery, which offers a system that significantly extends the service life of concrete structures, reducing maintenance costs. Microstructure tests also confirmed the results of the mechanical behavior.

## Materials and methods

**Fig. 1:**
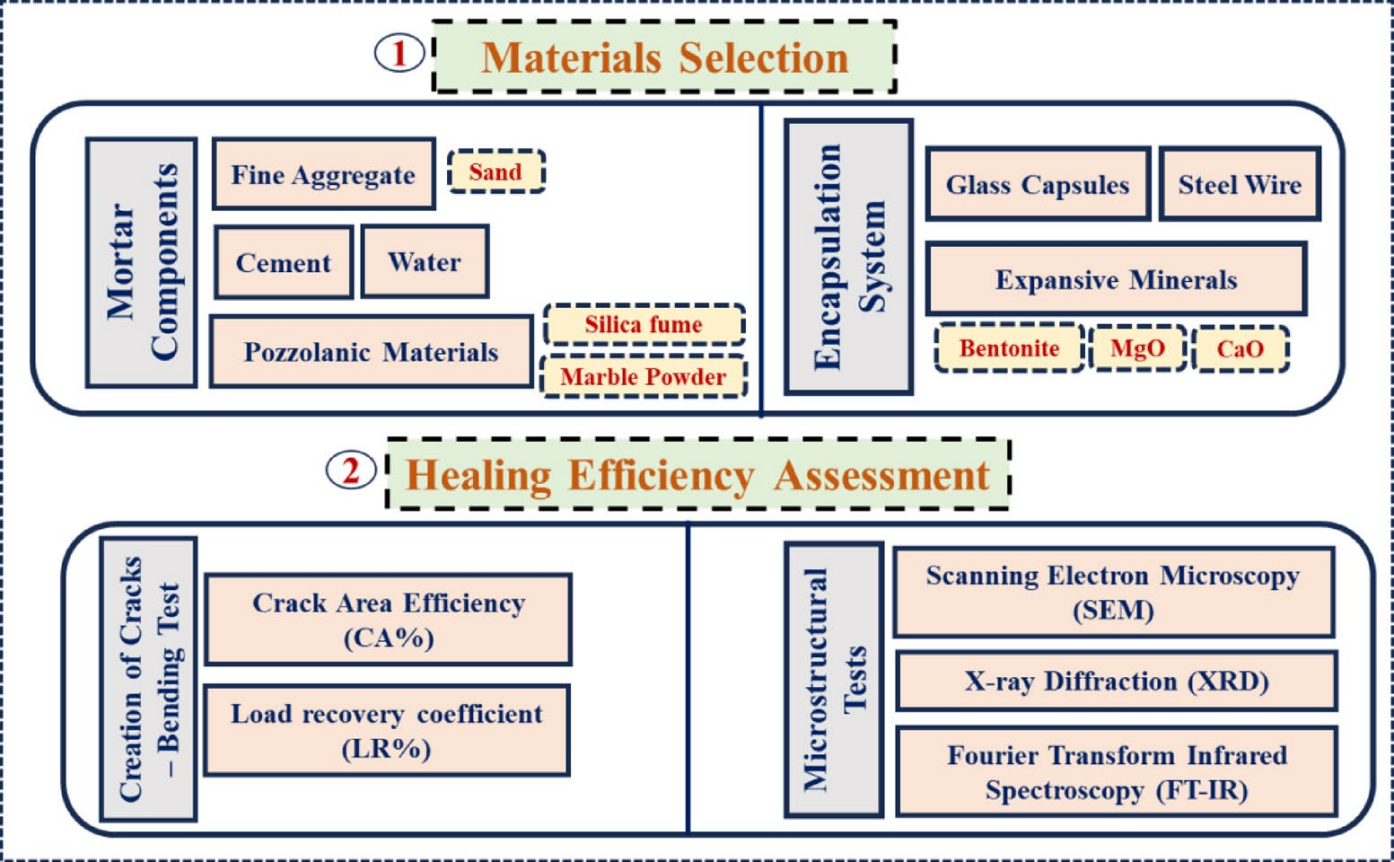
Schematic of the experimental program.

An experimental plan was designed to evaluate the healing efficiency using an encapsulation system in pozzolanic mortar, as illustrated in Fig. 1.

### Materials

Ordinary Portland cement (CEM-I 42.5 N) used in this study meets Egyptian standard specifications (E.S.S. 4756-1/2022)^48^. Laboratory tests confirmed that its chemical analysis and physical characteristics are suitable for mortar work. Clean tap water was used for the mixing process. The fine aggregate used in the mortar mixes was high-quality natural siliceous sand that was devoid of contaminants. Testing of the used fine aggregate met Egyptian Standards E.S.S. 1108/2021^49^: specific gravity 2.5, bulk density 1.52 t/m^3^, and fineness modulus 3.

This research used two types of pozzolanic materials: silica fume (SF) and marble powder (MP). The SF was supplied by Sika Egypt, while the MP marble powder was obtained from SGMC Company. The chemical compositions of these pozzolanic materials are presented in Table 1, and their physical properties are detailed in Table 2, based on the manufacturer’s data sheets.

**Table 1:** The chemical composition of the used pozzolanic materials.

Oxide composition	Percent by weight (%) *	
	Silica fume (SF)	Marble powder (MP)
Calcium oxide (CaO)	1.3	55.011
Silicon oxide (SiO_2_)	90.2	0.965
Aluminum oxide (Al_2_O_3_)	1.0	0.237
Magnesium oxide (MgO)	2	0.139
Ferric oxide (Fe_2_O_3_)	2.2	0.259
Sulfur trioxide (SO_3_)	0.3	0.090
Sodium oxide (Na_2_O)	0.7	0.071
Potassium oxide (K_2_O)	1.5	0.022
Insoluble residue	–	42.8
Phosphorus pentoxide (P_2_O_5_)	–	0.021
Strontium oxide (SrO)	–	0.30
Moisture	0.8	0.12

*By the manufacturer’s datasheet.

**Table 2:** The physical properties of the used pozzolanic materials.

Property	Result*	
	Silica fume (SF)	Marble Powder (MP)
Surface area (m^2^/kg)	17,000	4076
Particle size, µm	0.12	16
Specific gravity	2.20	2.84
Color	Gray	White

*By the manufacturer’s data sheet.

Expansive mineral compounds, including MgO, CaO, and Bentonite, were utilized in this study. Bentonite was obtained from RPMINERALS in Borg El Arab, Alexandria, Egypt. Reactive MgO and CaO were sourced from the Loba Chemie company. Their chemical compositions and physical properties are presented in Tables 3 and 4, respectively.

**Table 3:** The chemical composition of the used expansive mineral compounds.

Oxide composition	Percent by weight (%) *		
	Bentonite	MgO	CaO
Silicon oxide (SiO_2_)	61.03	0.89	0.88
Aluminum oxide (Al_2_O_3_)	14.59	0.22	0.12
Ferric oxide (Fe_2_O_3_)	2.09	0.48	0.07
Calcium oxide (CaO)	0.77	0.90	94.0
Magnesium oxide (MgO)	2.22	95.7	0.50
Sulfur trioxide (SO_3_)	0.37	–	0.08
Calcium carbonate (CaCO_3_)	–	–	3.7
Potassium oxide (K_2_O)	0.76	–	–
Sodium oxide (Na_2_O)	2.04	–	–
Chloride (CL^-^)	0.46	–	–
Titanium dioxide (TiO_2_)	0.22	–	–
Barium oxide (BaO)	0.11	–	–
Loss on ignition (L.O.I)	13.2	1.76	0.60

*By the manufacturer’s datasheet.

**Table 4:** The physical properties of the used expansive mineral compounds.

Property	Result*		
	Bentonite	MgO	CaO
Specific gravity	2.82	3.58	3.31
Grain size (μm)	4.75	4.20	3.30
Surface area (cm^2^/gm)	4800	–	–
Color	Light Yellow	White	off white

*By the manufacturer’s datasheet.

### Preparation of encapsulation system

**Fig. 2:**
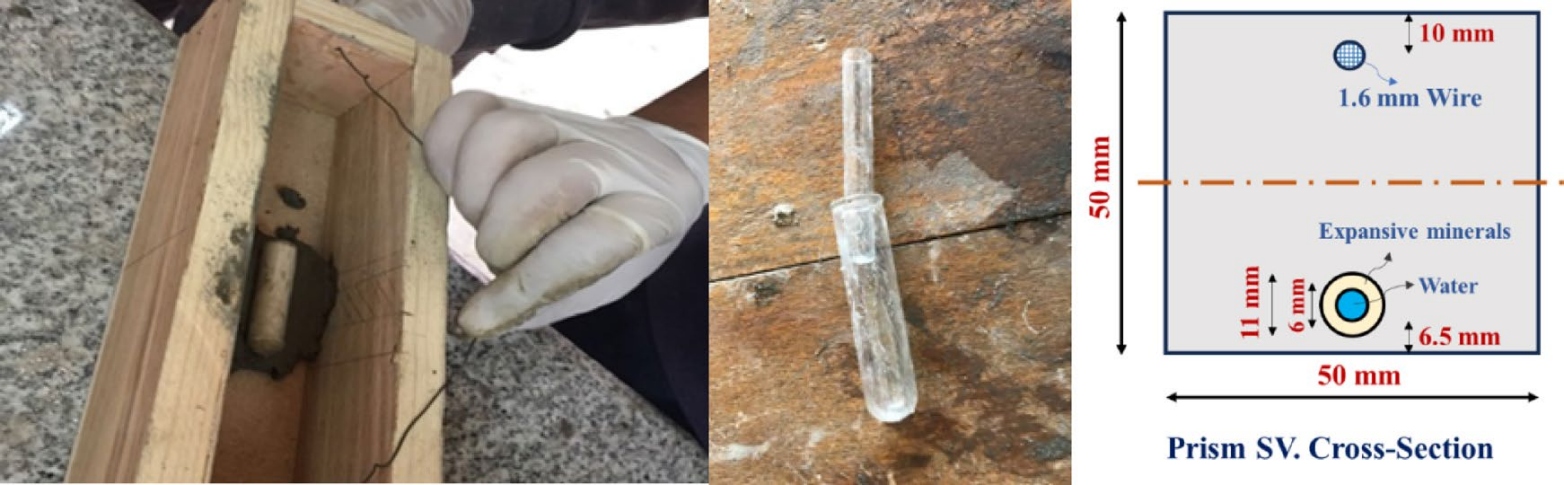
Encapsulation system details.

An encapsulation system consists of two soda glass capsules with a thickness of 0.45 mm, a length of 50 mm, and inner diameters of 6.15 mm and 11.4 mm for the inner and outer capsules, respectively as shown in Fig. 2. These capsules were embedded in 50 × 50 × 220 mm mortar prisms. An encapsulation system consists of two soda glass capsules with a thickness of 0.45 mm, a length of 50 mm, and inner diameters of 6.15 mm and 11.4 mm for the inner and outer capsules, respectively as shown in Fig. 2. These capsules were embedded in 50 × 50 × 220 mm mortar prisms. The inner capsule was filled with the expansive healing mixture. This mixture consisted of 40% MgO, 40% CaO, and 20% Bentonite of the volume of the capsule. The outer capsule was filled with water. The MgO and CaO powders used had an average particle size already given in Table 4. The expansive mineral powders used were in an anhydrous state. Sealing was achieved by applying three layers of 50 μm-thick PVC film, adhered using a rapid-curing, low-viscosity epoxy lacquer. The seal integrity was visually inspected before embedding. The center of the double-shell capsule system was aligned precisely with the midpoint of the prismatic beam’s length and placed 6.5 mm above the bottom face, which is the expected location of maximum tensile stress and primary crack initiation from the pre-cracking notch.

The 0.45 mm-thick soda glass capsules were designed to rupture at a critical crack opening displacement (COD) of 0.4 mm to 0.5 mm. This criterion ensures the release of healing agents upon the formation of microcracks. Rupture was verified during the initial loading cycle by the audible breakage sounds and the observance of water emerging on the prism faces. Post-mortem verification for selected samples was conducted via visual inspection of the fracture surface after the final flexural test. Furthermore, the successful release and reaction of the expansive agents were confirmed chemically via XRD and FT-IR detection of Brucite (Mg(OH)2) and CaCO3 in the healing products.

To prevent catastrophic damage to the samples during and after loading, a mild steel wire with a diameter of 1.6 mm was placed in the top half of ALL specimens (M1, M2, and M3), with a cover of 10 mm, i.e., placed in the compression zone. This wire served as secondary reinforcement to limit the maximum crack opening and maintain the required structural integrity for long-term healing monitoring. Because the wire was used consistently across all mixes, its constant mechanical contribution is factored out, allowing the differences in the measured Load Recovery (LR%) to be attributed solely to the effectiveness of the autonomous and autogenous healing agents. It is worth noting that since the wire was used in all specimens, its contribution to the final load recovery is present in all mixes. However, because it is placed far from the tensile face (where healing occurs) and is identical in all samples, it acts as a constant baseline mechanical strength. The differences in LR% between all mixes are therefore genuinely reflective of the healing efficiency of the healing agents (additives and capsules).

### Mix design, mixing, casting procedure, and curing

The mixture proportions were determined, and the weight of each component required to produce one cubic meter of mortar was estimated using the absolute volume method. Three different mortar mixes were created, as detailed in Table 5. The sand-to-cement ratio was 1.5:1 by weight in all mixtures, and the water-to-binder ratio was 0.42.

**Table 5:** The components of mortar mixes.

Mix	Cementitious materials %			Cementitious materials (kg/m^3^)			Water (kg/m^3^)	Sand (kg/m^3^)
	Cement %	SF %	MP %	Cement	SF	MP		
M1	100	0	0	797.97	0	0	319.19	1196.96
M2	85	15	0	785.10	117.76	0	314.04	1177.64
M3	85	0	15	794.68	0	119.20	317.87	1192.02

The mixing process for all mortar mixes followed this sequence: First, the calculated amounts of cement and sand were thoroughly mixed in a mechanical horizontal pan mixer for two minutes without water. For mixtures M2 and M3, fine silica fume or marble powder was then added and mixed at low speed for an additional two minutes. Next, water was added to the mixture and mixed for about five minutes until a uniform consistency was achieved. Finally, the fresh mortar was poured into molds in three layers, following ASTM C-192/C192M^50^, with each layer compacted using a vibrating table for 30 s. The specimens were demolded one day after casting and cured in tap water until test time. There were three replicas of each case of the tested specimens.

### Creation of cracks and mechanical loading

**Fig. 3:**
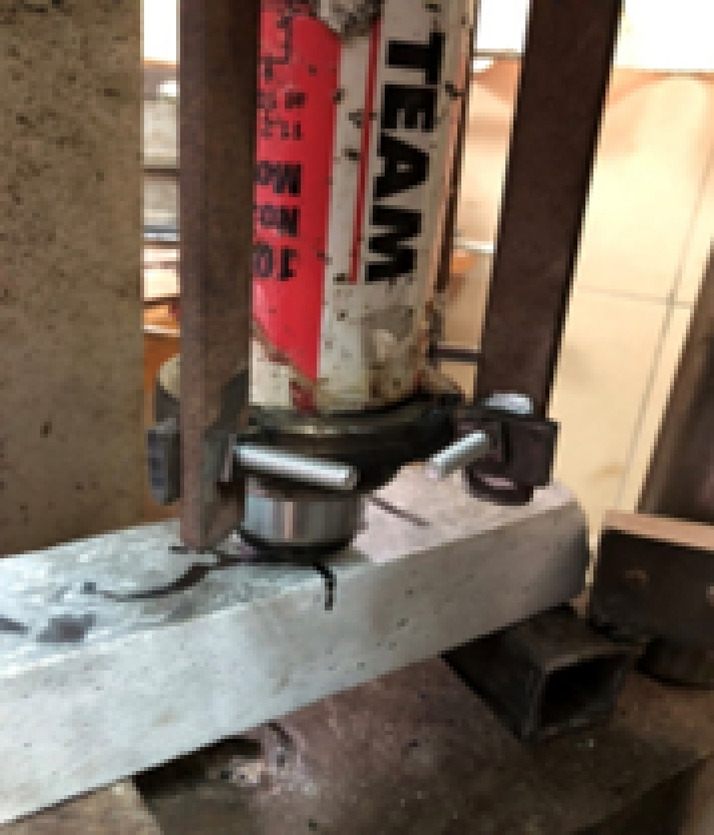
Bending test setup.

Before loading, the prism samples were notched with a rotating diamond blade, featuring a notch depth of 1.5 mm and a width of 2.0 mm. The samples were then subjected to a three-point bending test using a 30 kN static loading frame (Fig. 3) with a support span of 200 mm; the load was applied at a displacement control mode at a rate of 0.125 mm/min. The support and loading platens were meticulously aligned to ensure the load was applied centrally, maintaining a lateral alignment tolerance of less than 1% of the specimen width. The clip gauge was calibrated to a measurement uncertainty of ± 0.005 mm, and loading was halted when the cracks opened to 0.40–0.50 mm, which allowed the glass capsules to rupture and release the healing agent. Upon full load removal, the crack width immediately defined the initial crack area (A0), with each specimen exhibiting an average residual crack width in the range of 0.36–0.38 mm. Subsequent measurements of the residual crack area (At) were performed manually using a graduated lens/microscope (with a precision of ± 0.01 mm) at three fixed, marked points (Point 1, Point 2, and Point 3) along the crack path to ensure the repeatability of the crack area efficiency (CA%) calculations. After cracking, the three samples representing each mix type were allowed to heal by immersing them in water at a constant temperature of 20 ± 1 °C for 28 days. The mean value of each set of results was calculated in addition to the coefficient of variation percentage (CV%), CV% = (standard deviation/mean) × 100.

It is worth noting that continuous water immersion at about 20 ± 1 °C throughout the 28-day healing period was adopted in the present study as it represents the conventional and standardized curing method. This method was intentionally adopted to evaluate the maximum achievable self-healing potential of our composite system in an ideal, supportive environment. However, the effect of the curing condition plays a crucial role in self-healing efficiency, as found by Qureshi et al.^46^.

### Healing efficiency

#### Crack area efficiency (CA%)

**Fig. 4:**
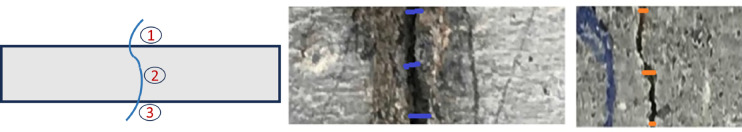
Surface crack width measurement.

Surface cracks were intersected by three parallel lines, with the intersections marked as C1, C2, and C3, as shown in Fig. 4. The widths of the cracks were measured using a graduated lens. Measurements were taken after 3, 7, 9, 12, 15, 18, 21, 25, and 28 days of healing. The crack area efficiency (CA%) was calculated for each measured crack width. The reduction in crack opening area over time, referred to as crack sealing area efficiency, was determined. The CA% was calculated using Eq. 1^46^.

1 $$CA\%= \frac{{A}_{0}-{A}_{t}}{{A}_{0}}\times 100$$

where: CA%: Crack Area Efficiency. A_0_ (mm^2^): The Initial Crack Area immediately after cracking, before healing (Positive Area). A_t_ (mm^2^): The Residual Crack Area measured after the healing period at time t (Positive Area).

#### Load recovery coefficient (LR%)

The load recovery coefficient (LR%) can be calculated by Eq. 2:

2 $$LR\%= \frac{{P}_{max,h}-{P}_{i}}{{P}_{max}-{P}_{i}}\times 100$$

where P_max,h_ (N): The maximum load attained by the healed specimen during the reloading flexural test (Positive Load). P_i_ (N): The initial pre-cracking load is applied to create the crack before the healing period begins (positive load). P_max_ (N): The maximum peak load achieved by the uncracked reference specimen (Positive Load).

### Microstructural tests

To analyze the microstructure of mortar and healing materials, mortar mixes were examined using scanning electron microscopy (SEM), X-ray diffraction (XRD), and Fourier transform infrared spectroscopy (FT-IR). Samples were obtained from the innermost core of crushed specimens following the bending strength test at 28 days of age.

For SEM analysis, an electron microscope (JEOL JSM-6510LV) with magnification capabilities up to 300,000 times was utilized at the Faculty of Agriculture, Mansoura University, Egypt. Observations were conducted at three magnifications: 100X, 750X, and 1500X. Before imaging, samples were dried at 80 °C and coated with a thin coating layer, and the observations were conducted using an accelerating voltage of 30 kV. XRD analysis was performed using a Philips X’Pert Pro MPD PW 3050/60 X-ray diffractometer at the Housing and Building National Research Center (HBRC). Room-temperature powder X-ray diffraction with a 0.154 nm filter was employed for the analysis.FT-IR analysis was conducted using a Jasco-6100 spectrometer with samples prepared as KBr binders at HBRC. Samples for FT-IR analysis were carefully collected and ground to pass through a 75 µm sieve.

## Results and discussion

During the first round of testing, as the load peak was reached, two distinct breakage sounds were heard. The hissing noise indicated that the two glass capsules within the prisms had ruptured. Upon rupture, water was observed on the side and bottom faces of the prisms, emerging as the capsules were breached. The samples were fully fractured in the second testing cycle, and the crack opening widened to 0.5–0.6 mm. The additional liquid was released from the embedded capsules, suggesting that some healing compounds remained inside the capsules after the initial rupture. This residual healing compound may facilitate a second round of healing in response to larger crack sizes.

### Surface crack width and crack area measurement

**Fig. 5:**
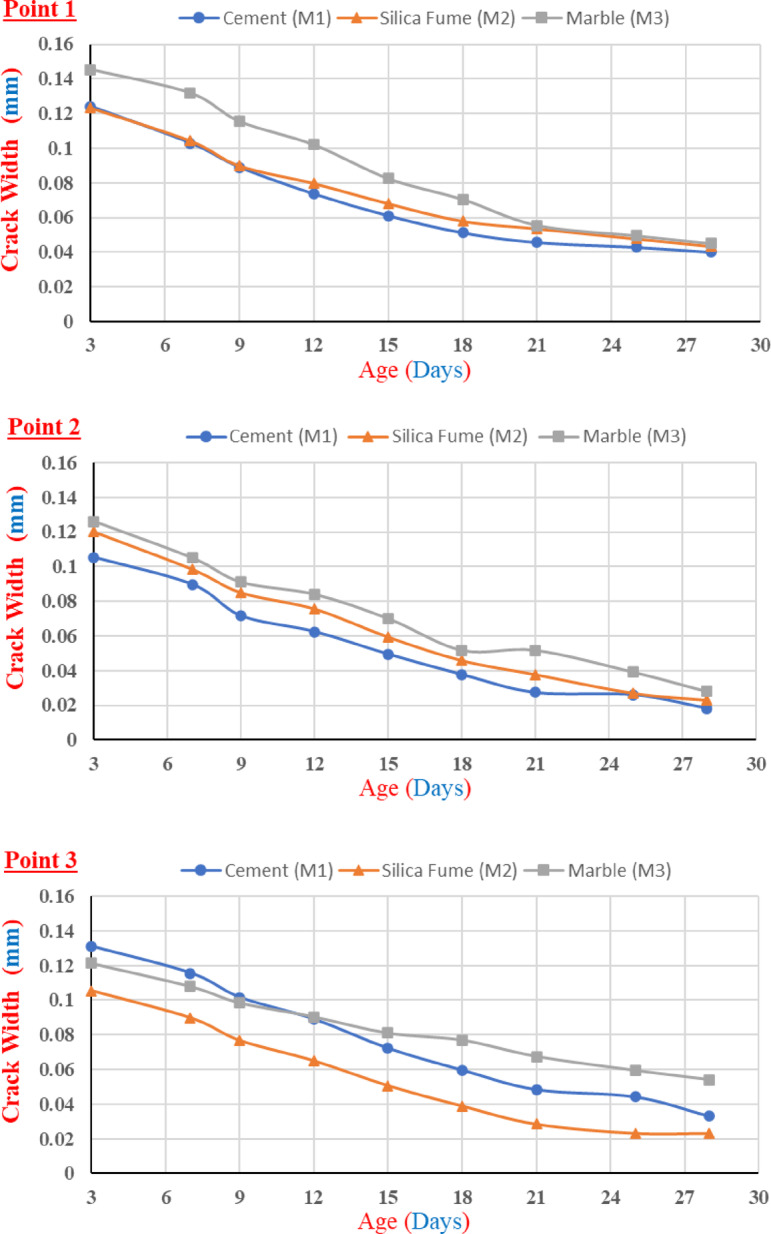
The change in crack width with time.

Figure 5 shows the measured crack width versus time (age). It was found that the width of the crack surface decreased with time due to the healing resources filling its surface. As stated by Jiang et al.^52^, these resources grew from both sides of the crack toward the middle. While the crack width reduction shown at individual points indicates effective sealing by all mortar types, the overall healing performance was primarily quantified using Crack Area efficiency (CA%) and load recovery (LR%). Since silica fume produces CSH gels than other materials, this can be attributed to the fact that silica-based materials (such as silica fume) are more efficient at healing the cracks by expansion of the healing resources, which fill the cracks with crystals^51,52^.

The assessment of crack width measurements across the full 3- to 28-day curing period and at three distinct locations (Point 1, Point 2, and Point 3) confirmed a high degree of self-healing consistency across all mixtures, as the CV% values consistently ranged only between 8.0% and 16.0%. This analysis established a clear performance hierarchy where silica fume (M2) consistently outperformed marble powder (M3), which in turn was more effective than cement (M1) in promoting crack sealing. The CV% ranges were quantified sequentially: at Point 1, cement (M1) exhibited the highest variability (13.0% to 16.0%), followed by marble powder (M3) (11.5% to 12.8%) and silica fume (M2) (8% to 11.5%). Point 2 showed the fastest self-healing efficiency, correlating with the lowest CV% ranges across all materials at that location, with cement (M1) ranging from 12.0 to 13.5%, silica fume (M2) demonstrating the absolute lowest variability (10.0% to 10.8%), and marble powder (M3) ranging from 11.0 to 12.0%. Finally, the consistency trends were upheld at Point 3, where cement (M1) ranged from 12.8% to 13.8%, silica fume (M2) ranged from 10.5% to 11.2%, and marble powder (M3) ranged from 11.3 to 12.5%. This detailed consistency analysis confirms that the silica fume mixture possesses the most robust and predictable self-healing mechanism, resulting in superior performance and the lowest measurement variability across all points.

**Fig. 6:**
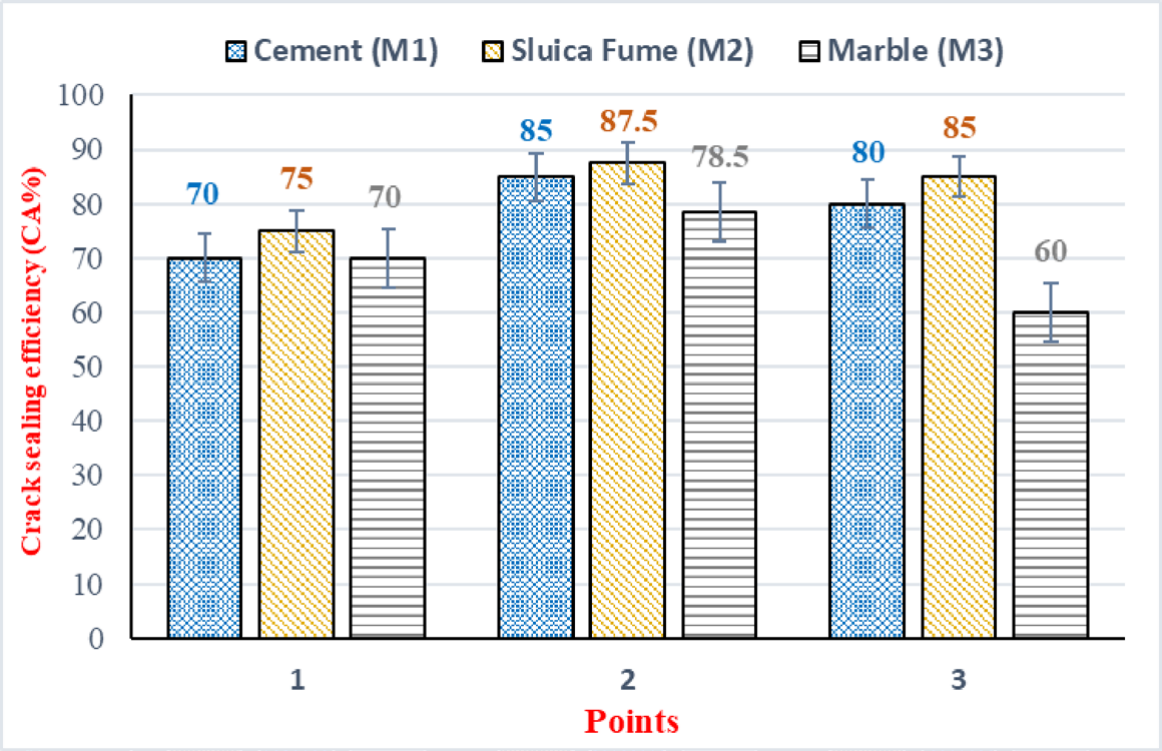
Crack sealing efficiency (CA)% at 28 days.

**Fig. 7:**
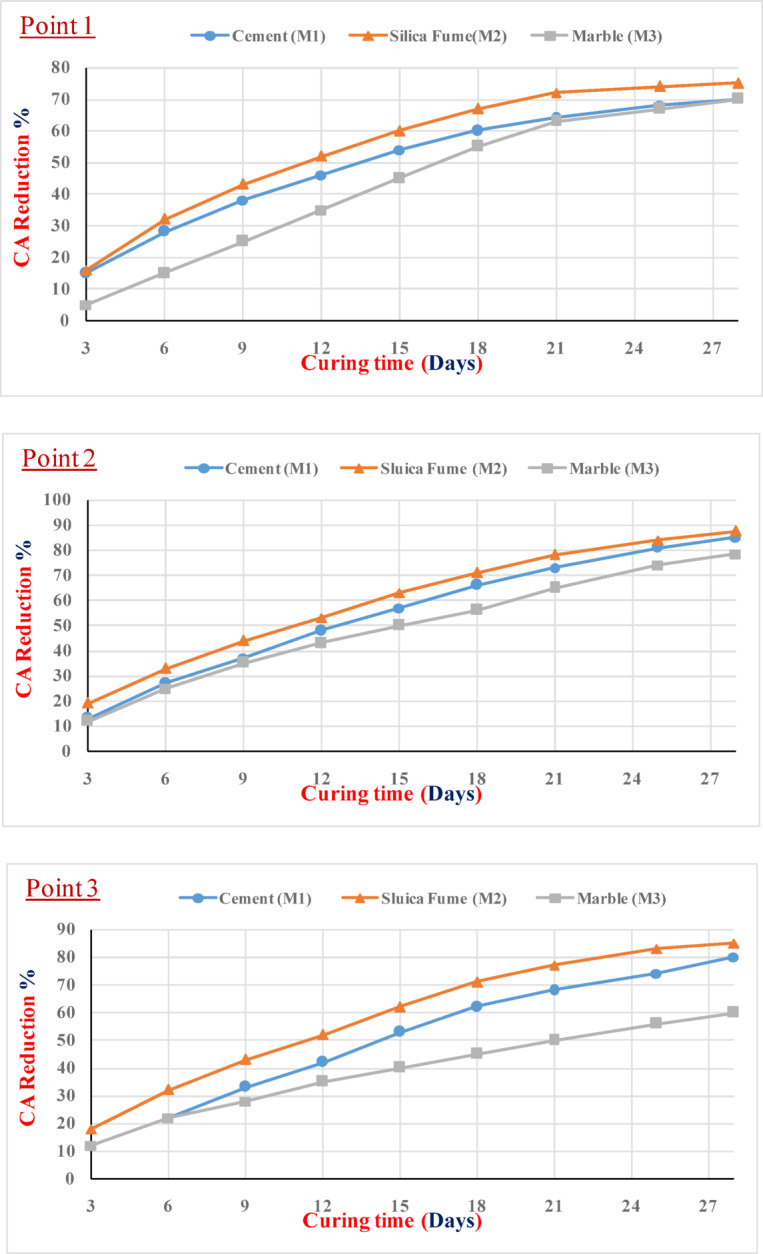
CA reduction % at different ages.

The crack sealing efficiency at the three distinct points across the three sample types is illustrated in Fig. 6. Three samples are included in each type. At point 1, samples incorporating silica fume (M2) demonstrated a sealing efficiency of 75% after 28 days. In contrast, both the marble-containing samples (M3) and the control mixes exhibited the lowest sealing efficiency, with a value of 70% at point 1 after 28 days. At points 2 and 3, the silica fume (M2) samples exhibited the highest sealing efficiency, achieving 87.5% and 85%, respectively. These values surpassed those of the control samples, which reached 85% and 80%, respectively. Furthermore, the marble-containing samples (M3) again exhibited the lowest crack sealing performance at both points, with values consistently lower than those of the M2 and control samples after 28 days. Furthermore, the crack sealing efficiency as a function of curing time for the three distinct points across the three sample types is illustrated in Fig. 7.

The evaluation of Crack Sealing Efficiency across the three measurement points (Point 1, Point 2, and Point 3) confirmed a clear performance hierarchy: silica fume (M2) consistently achieved the highest sealing efficiency, followed by cement (M1), with marble powder (M3) being the least efficient. The CV% was quantified at each location: at Point 1, cement (M1) recorded a CV% of 13.5%, silica fume (M2) recorded 7.0%, and marble powder (M3) recorded 13.8%. Point 2 correlated with the highest CA% performance and the lowest overall variability, where cement (M1) recorded a CV% of 12.5%, silica fume (M2) recorded the lowest CV% in the study at 6.5%, and marble powder (M3) recorded 13.0%. Finally, at Point 3, cement (M1) recorded 13.0%, silica fume (M2) recorded 7.5%, and marble powder (M3) recorded the highest variability at 13.9%. This robust analysis confirms that the silica fume mixture offers a highly reliable and stable self-healing mechanism, resulting in superior CA% and minimal measurement variability across all points. Also, the evaluation of Crack Area Reduction Percentage across the full curing period and the three measurement points established clear distinctions in mixture performance and measurement variability. The CV% values supported this hierarchy: At Point 1, cement (M1) recorded the highest variability with a CV% of 13.4%, marble powder (M3) recorded 11.5%, and silica fume (M2) recorded 10.0%. Point 2 showed the fastest overall reduction rate, correlating with the lowest CV% values across all materials at that location: cement (M1) recorded 12.5%, silica fume (M2) recorded the absolute minimum CV% of 8.0%, and marble powder (M3) recorded 10.5%. Finally, at Point 3, cement (M1) recorded 13.0%, silica fume (M2) recorded 9.5%, and marble powder (M3) recorded 11.2%.

### Images of the cracked and healed surfaces

**Fig. 8:**
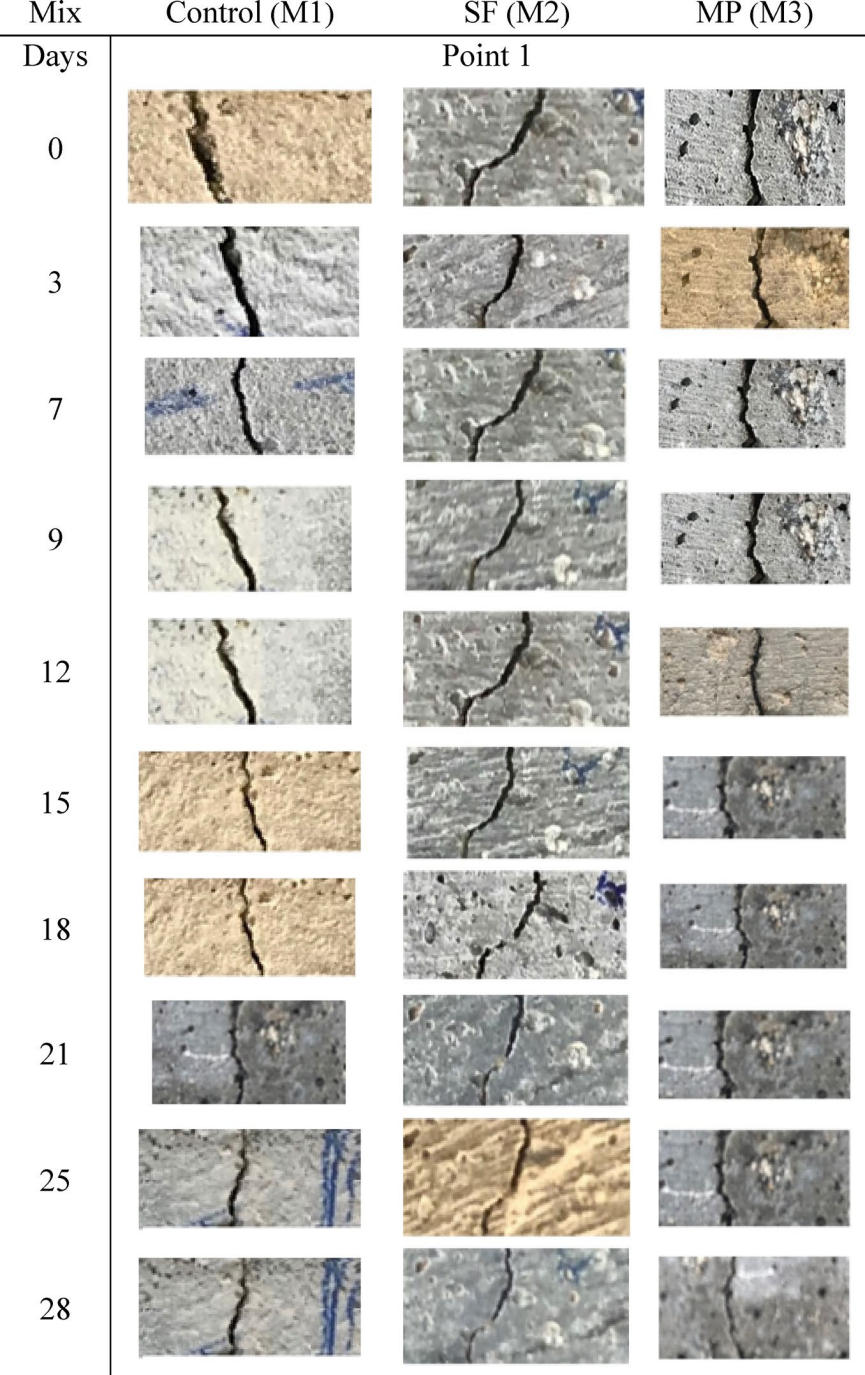
Images of the cracked and healed surfaces for the three mixes at different ages.

At the ages of 0, 3, 7, 9, 12, 15, 21, 25, and 28 days, photographs of the three mortar mixes with various crack widths show images of the artificial crack. Figure 8 demonstrates that crack width diminishes over time for all three materials. Each type consists of three samples. However, marble consistently exhibits the widest cracks, and its reduction rate with curing is less pronounced compared to cement and silica fume.

### Load recovery

**Fig. 9:**
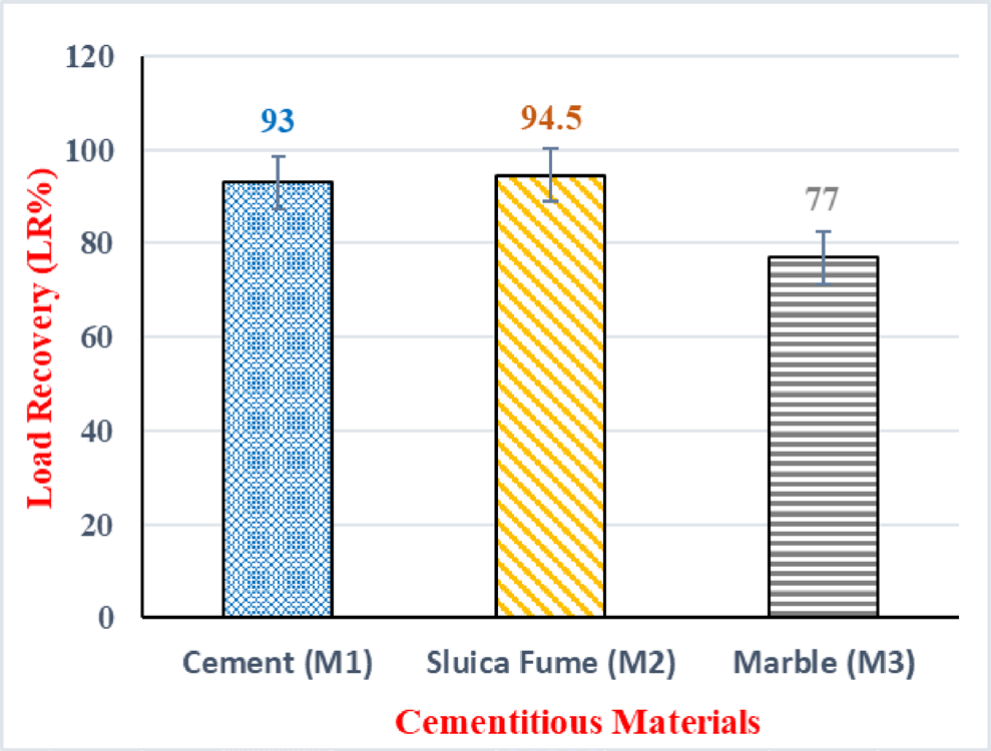
Load recovery (LR%) at 28 days.

When the mechanical strength recovery of the healed specimens was analyzed, the recovery values revealed patterns similar to each sample’s crack healing effectiveness. The crack healing effectiveness was quantified based on the reduction in crack width from the Initial Crack Area (A0) to the Residual Crack Area (At), as defined by the Crack Area Efficiency (CA%) (Eq. 1). It was also found that the minerals in the capsules had moistened and crystallized over time. Significant mechanical strength recovery was triggered by the hardened capsules acting as a bridge between cracks^53^. Figure 9 displays load recovery (LR%) at 28 days. During reloading, prisms were totally broken, and it was observed that cracks reopened in the same places. The encapsulation’s released minerals began to hydrate as soon as the crack appeared, and as time went on, the healing materials began to precipitate. At the crack zone’s air/water interface cross-section, carbonate-rich self-healing products—specifically, calcite and calcite combined with other hydration compounds—accumulated and were visually recognized as superficial healing materials^54^. When comparing the SF samples to the others, a significant improvement in mechanical recovery was observed for the SF samples. At 28 days, load recovery results ranged from 15% for MP samples to 20% for the SF samples, with the load recovery trend following the sequence: M2, M1, and M3. This indicates the higher load recovery performance of mixM2 samples compared to both mix M1 and M3.

The high Load Recovery (LR%) seen in all mixes, notably the SF mix, which achieved 20% recovery, is due to the synergistic impact of the autogenous healing (pozzolanic activity) and the autonomous healing component given by the macro-capsules. When a crack forms, the encapsulated expansive minerals (MgO, CaO) and bentonite are released, causing a localized mechanical pressure and volume increase. This provides the necessary expansive force to effectively bridge the crack and restore the beam’s structural load-bearing capacity, validating the encapsulated system’s key function. The overall consistency of these load recovery measurements was quantified using the CV% values, which all remained within a range of 8.0% to 13.2%. Supporting its superior performance, the silica fume (M2) mixture demonstrated the lowest measurement variability, with a CV% of approximately 8.0%, indicating the most reliable mechanical self-healing. The cement (M1) mixture showed moderate consistency, recording a CV% of roughly 10.5%, while the marble powder (M3) mixture, which registered the lowest LR%, also exhibited the highest measurement variability, with a CV% of approximately 13.2%. This analysis confirms that the inclusion of silica fume results in the most effective and stable mechanical self-healing capability.

### SEM results

**Fig. 10:**
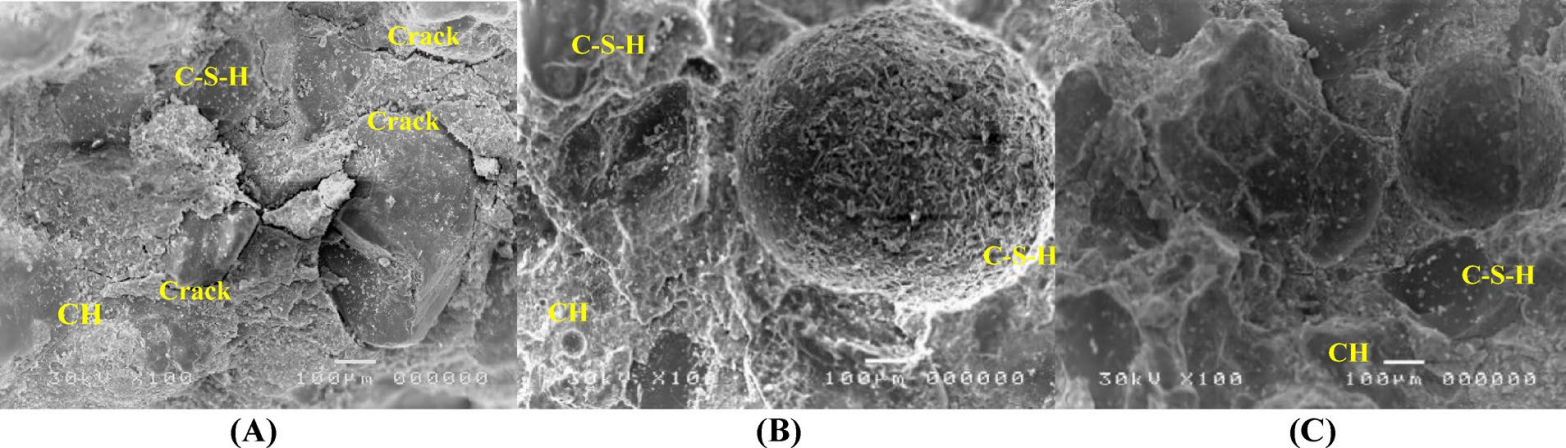
SEM images (100X) after 28 days of curing for mixes (**A)** M1, (**B0** M2, and **C** M3.

**Fig. 11:**
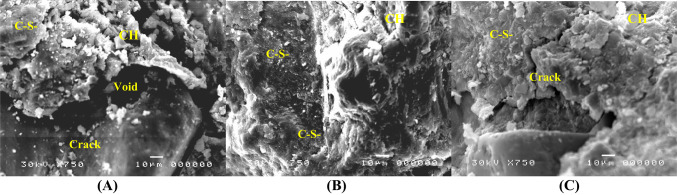
SEM images (750X) after 28 days of curing for mixes (**A)** M1, (**B)** M2, and (**C)** M3.

**Fig. 12:**
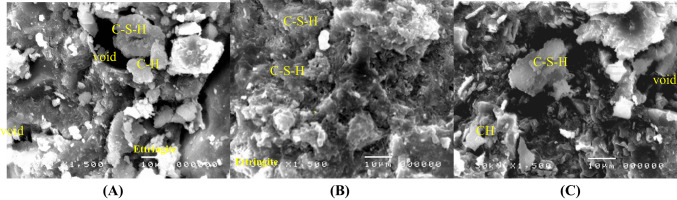
SEM images (1500X) after 28 days of curing for mixes (**A)** M1, (**B)** M2, and (**C)** M3.

The results of SEM are presented in Figs. 10, 11 and 12. The results indicated that adding silica fume to mix M2 significantly improved the microstructure of the mortar matrix, making it denser with reduced voids compared with mix M1. Also, silica fume enhanced hydration, resulting in the development of a cohesive calcium-silicate-hydrate (C–S–H) gel and a decrease in calcium hydroxide (CH) content. This results in a more refined interfacial transition zone (ITZ) with improved mechanical characteristics and mortar durability. The bond between the aggregate and cement paste was strengthened due to SF’s higher-fineness particles, which efficiently filled the matrix’s pores^55^. However, marble powder had a less significant effect on the microstructure. However, when comparing the influence of SF and MP, it was found that MP can fill pores and enhance the hydration process, but not with the efficiency of SF^56^. Overall, SF had a significant advantage in terms of improving mortar microstructure and ITZ quality.

The SEM analysis confirmed the activation and function of the autonomous healing system. Upon cracking, the macro-capsules ruptured, releasing the expansive mineral agents (MgO/CaO) and bentonite. Figures 10, 11, and 12 provide microstructural evidence of this release by showing the presence of dense, newly formed healing products inside the crack volume. These products are visibly different from the surrounding mortar matrix, appearing as masses of CaCO₃ crystals and highly localized Mg-rich hydration products (Mg(OH)₂ or M-S–H phases). This direct visual confirmation demonstrates that the encapsulated minerals successfully triggered the expansive healing mechanism crucial for structural load recovery (LR%).

### X-ray diffraction results

**Fig. 13:**
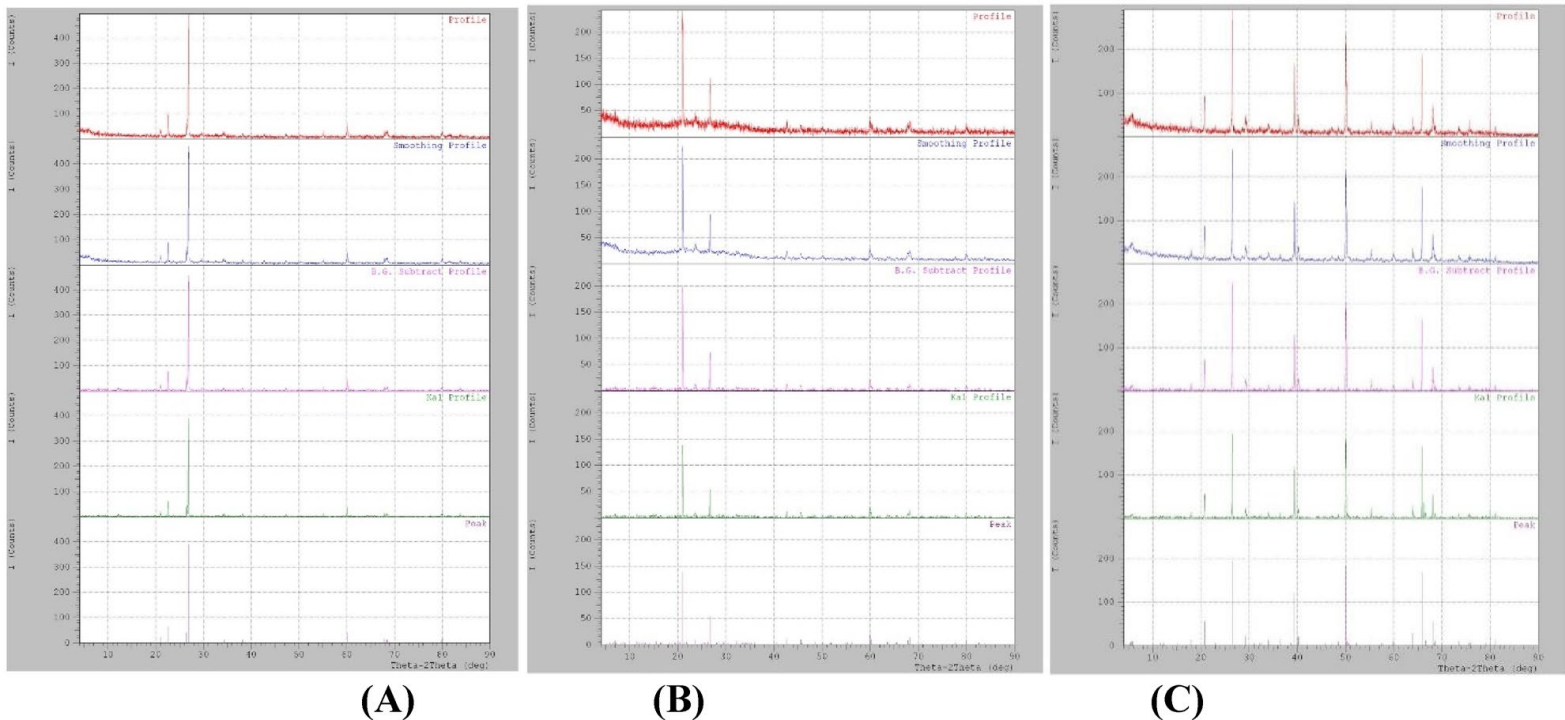
X-ray diffraction after 28 days of curing for mixes **A** M1, **B** M2, and **C** M3.

The results of the XRD test for the three mortar mixes show that the mixes have distinguishable phase compositions, which change with the addition of silica fume and marble powder, as shown in Fig. 13. For mix M1, without pozzolanic material, calcium hydroxide (CH), calcium silicate hydrate (C–S–H), and ettringite are the primary hydration products because there are no reactive additives. Also, observing the result of Mix M2 shows a lower CH content and more amorphous hydration products due to silica fume promoting pozzolanic reactions, which improve the microstructure of the matrix and the formation of C–S–H^57^. Furthermore, the calcite peaks that were observed in the result of mix M3 were due to the utilization of marble powder. Marble does not participate in pozzolanic action; consequently, the CH content is comparable to or a little higher than in mix M1, mainly acting as a filler for enhancing the mechanical properties of the mortar^58^. The results of the XRD test showed that the addition of expansive materials, MgO, CaO, and bentonite, influenced the matrix crystalline phases. In all tested mortar mixes, a significant peak of brucite (Mg(OH)₂) was observed, which was formed by Mg^59^.

However, as CaO hydrates to calcium hydroxide, the probability of CH peaks existing in Mixes M1 and M3 increases. Even so, because of the pozzolanic reaction with silica fume, Mix M2’s CH peak intensities are lower than those of M1 and M3^60^. The verified presence of Mg(OH)₂ confirms the successful release and response of the encapsulated mineral agents. The development of this expansive product is crucial because the increased volume provides the mechanical self-prestressing required to achieve the high Load Recovery (LR%), demonstrating the significance of the autonomous healing component.

### FT-IR results

**Fig. 14:**
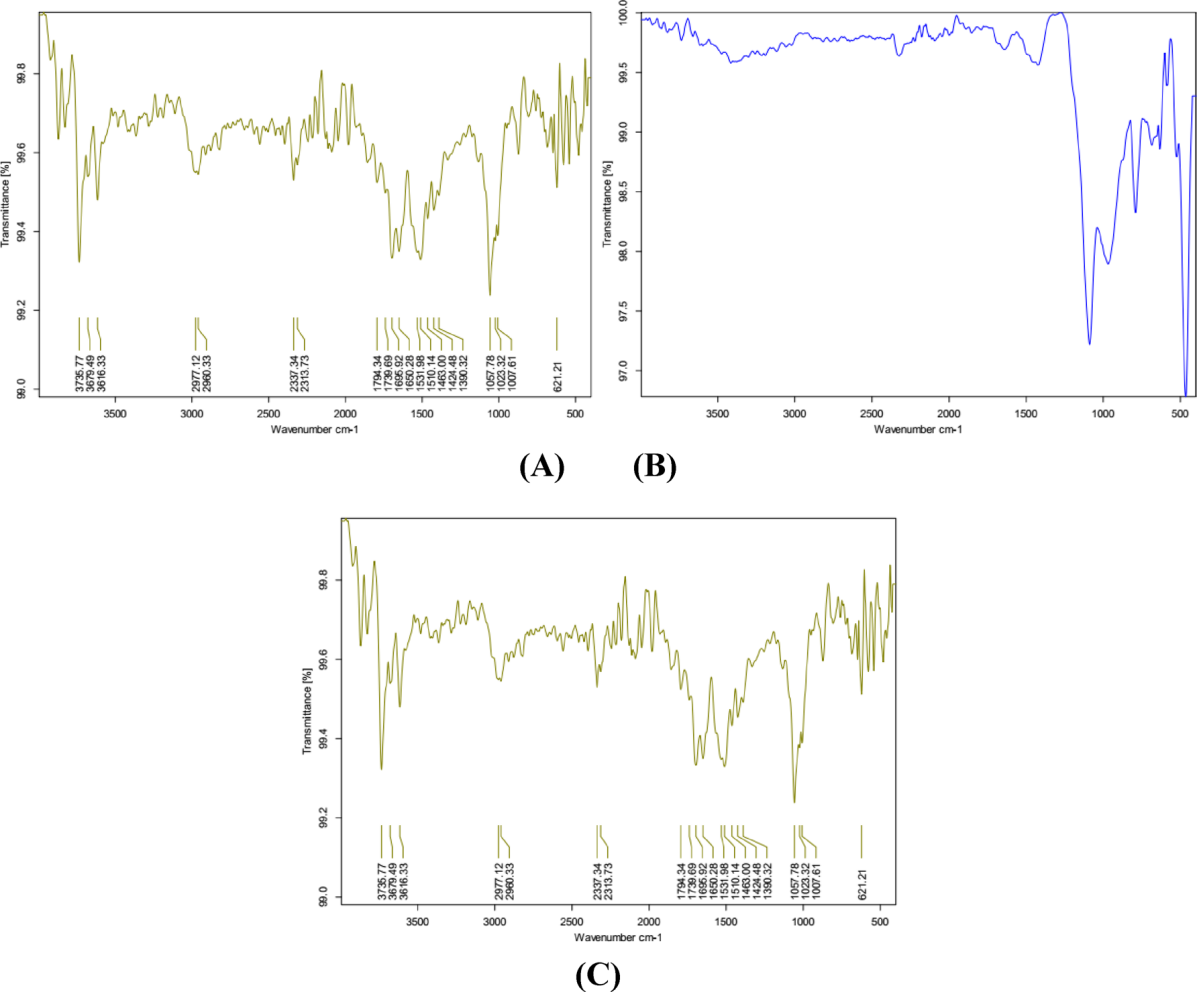
FT-IR after 28 days of curing for mixes **A** M1, **B** M2, and **C** M3.

The effect of adding cementitious and expansive materials on the chemical composition of the tested mixes is studied by the results of FT-IR, which are presented in Fig. 14. Without cementitious materials, the usual products of hydration, calcium hydroxide (CH), calcium silicate hydrate (C–S–H), and water-related vibrations are exhibited for mix M1. However, due to the pozzolanic reaction of fine SF particles, mix M2 shows a significant decrease in CH content, which advances the formation of additional C–S–H and improves the microstructural properties of the mortar matrix^61^. Also, as a result of adding MP, it was observed that Mix M3 offers strong calcite-related signals without a significant decrease in CH or an increase in C–S–H content^62^.

In general, the results of Mixes M2 and M3 indicate that using SF is more effective in the hydration process activity, while using MP acts as an inert filler and affects the composition of mortar without introducing significant chemical reactivity^63–65^. The FT-IR study reveals that the autonomous healing mechanism was successfully activated by detecting the resultant chemical compounds. Specifically, a distinct peak matching the stretching vibration of the OH bond in brucite (Mg(OH)₂) was seen across all tested combinations, confirming the hydration and release of the encapsulated MgO. Furthermore, increased peak intensities for calcite (CaCO₃) in the healed fracture samples show that the CaO component also easily reacts. This chemical proof of expansive products (brucite and calcite) is critical since the volume expansion they give is the primary mechanism for obtaining high load recovery (LR%) and proving the operation of the encapsulated minerals.

## Conclusion

This study investigated the encapsulation of expansive mineral compounds (MgO, CaO, and bentonite) as healing materials for three cementitious material types. Cement mortar, silica fume, and marble mortar were used, and their self-healing performance was compared with respect to load recovery, surface crack width, and crack sealing efficiency. The following conclusions can be made.

1. Silica fume specimens exhibited the highest structural recovery, achieving 20% load recovery (LR%), outperforming marble samples (15%). This superior performance results from the synergical effect between enhanced pozzolanic C–S–H formation and the expansive, mechanical contribution of the encapsulated mineral agents (Brucite and Calcite) to crack-bridging.
2. Point 2 for all mixes showed the fastest self-healing over points 1 and 3.
3. Aluminosilicate, C–S–H, Mg(OH)₂, and Ca(OH)₂ hydrated and expanded in the crack surface and voids zone around it when the capsules were released.
4. The reactive hydrating products react with CO_2_ from water or atmospheric sources, forming different bicarbonates and carbonates, which are responsible for bridging cracks and causing the crack sealing, which considerably improves the strength.
5. Microstructural analysis confirmed the successful activation and release of the encapsulated minerals, with the accumulation of expansive Ca(OH)2 and Mg(OH)2 (Brucite) leading to denser healing compounds.
6. FT-IR analysis demonstrated that the silica fume’s pozzolanic activity through CH consumption and C–S–H formation, while marble powder primarily exhibited calcite peaks without enhancing hydration reactivity.
7. The expansive products, particularly Brucite (Mg(OH)2) derived from the capsules, along with C–S–H and Ca(OH)2, hydrate and expand, providing the volume stability required for crack sealing and structural load recovery.

## Data Availability

The authors declare that the data supporting the findings of this study are available within the paper.
